# Characteristics and patterns of individuals who have self-harmed: a retrospective descriptive study from Karachi, Pakistan

**DOI:** 10.1186/s12888-022-04018-7

**Published:** 2022-05-31

**Authors:** Ambreen Tharani, Salima Farooq, Maryam Pyar Ali Lakhdir, Uroosa Talib, Murad Moosa Khan

**Affiliations:** 1 School of Nursing and Midwifery, Aga Khan University, Second Floor, Stadium Road, P.O Box 3500, Karachi, Pakistan; 2 Department of Community Health Sciences, Aga Khan University, Karachi, Pakistan; 3 Karwan-E-Hayat, Psychiatric Care and Rehabilitation Center, Karachi, Pakistan; 4 Department of Psychiatry, Aga Khan University, Karachi, Pakistan

**Keywords:** Characteristics, Intent to die, Psychiatric illness, Self-harm, Suicide

## Abstract

**Background:**

Self-Harm (SH) is a major global public health problem under-researched in Pakistan due to religious and legal implications. This study aims to identify the characteristics and patterns among patients with SH and factors associated with the intent to die.

**Method:**

This retrospective descriptive study where SH cases presented to private tertiary care teaching hospital in Karachi, Pakistan, from January 2013 to December 2017 were extracted from HIMS records. Details related to demography, history, associated factors, access to methods used, and intent to die were collected on a structured proforma and analysed using STATA version 14.

**Results:**

A total of 350 cases were analysed. More than half of the reported cases were in the age group 20–39 years. Though only one-fourth of the SH cases had a past psychiatric history, it was found to be significantly (*P*-value < 0.05) associated with intent to die. Notably, 81% of the cases do not have a history of SH. Drug overdose (61.6%) and insecticides (36.6%) were the two most common methods used. Depression was identified in nearly half of the cases. The most common reason for attempting SH was inter-personal relationship issues (54.3%).

**Conclusion:**

This paper provides recent data on the characteristics and patterns associated with the intent to die of individuals who have self-harmed. In most cases of SH, past psychiatric history was not evident. Current psychiatric diagnosis and young adults were favoured in this study. The data from this study has limited representation for all demographic representation of SH cases from Pakistan as being from a single private hospital. There is a need for further research on SH in Pakistan.

**Supplementary Information:**

The online version contains supplementary material available at 10.1186/s12888-022-04018-7.

## What this paper adds

- Identify the most common cases reported with SH aged between 20–39 years.
- Past Psychiatric history and previous attempts were less reported in this study.
- Interpersonal conflicts were found as the more prevailing reason in SH cases.
- Benzodiazepines and pesticides were the most dominant methods used by both sexes.

## Background

Suicide is a major public health concern worldwide, irrespective of ethnicity, sex, age, culture, and religion [1]. Nearly 700,000 people annually die by suicide globally [2]. Approximately 79% of the global total reported cases of suicides are from low and middle-income countries (LMICs) [3]. A prior act of self-harm (SH) is one of the strongest predictors of future suicide in higher-income countries (HIC) [4, 5]. Research has shown that approximately 45% of people contacted their health care professionals about a month prior to the SH act; however, the characteristics and patterns of the SH act were mostly overlooked, and the necessary support was not provided [6, 7]. Literature from HIC also affirms that the risk of repeating SH after an index attempt increases in the future [5].

Pakistan is an Islamic country with an estimated population of 212 million, in which health and mental health care systems and related monitoring have lagged behind due to political and financial instability [8, 9]. Issues related to mental health receive less priority since the available resources are insufficient to meet the demands of other physiological health care needs.

Karachi is the country’s largest city, with an estimated population of approximately 22 million. It has the principal seaport and is the major industrial and commercial hub; hence, it attracts people from all parts of Pakistan. Therefore, it is a densely populated, multilingual, and multicultural city as Pakistan has a distinct variety of languages and cultures. It is estimated that about half of the population in the city lives in slums, with compromised access to basic necessities [10].

Over the last couple of decades, incidences of suicide and SH have gradually increased in the country [11]. However, both are underreported due to religious, legal, social, cultural, and moral taboos. In Pakistan, suicide and SH have been criminalized acts subject to legal punishment of imprisonment for a year and/or a fine [12]. The criminalization of suicide and SH are based on strong condemnation in Islam. Suicide is a preventable cause of death, and prior history of SH is a strong predictor of future suicides, as evident from studies from HIC [5]. However, there is a scarcity of data from the context of Islamic countries such as Pakistan. Therefore, studying the characteristics and patterns of SH is important for both primary and secondary prevention of suicidal behaviours (suicide and SH) in Pakistan. Identifying possible methods used for SH and their accessibility is essential. This study aims to identify the characteristics and patterns among patients with SH and factors associated with the intent to die presented during 2013–2017, at a private tertiary care teaching hospital in Karachi, Pakistan.

## Methods

A retrospective descriptive study was conducted to collect information about the characteristics and patterns of individuals who have presented with SH at the Aga Khan University Hospital (AKUH), Karachi, Pakistan, between January 2013 to December 2017. AKUH is a private, fee-for-service, tertiary care teaching hospital located in the centre of Karachi, with a capacity of about 700 beds. It has facilities for nearly all medical specialities, including an inpatient psychiatry unit with 18 beds. There is a 24-h psychiatry coverage provided for all general wards (including the emergency room) of the hospital as required. The hospital does not have a defined catchment area, and patients living anywhere in the city can access it. More than half of the patients seeking medical care belong to the low to middle socio-economic class and are from various parts of the country [7]. Records of all patients visiting the AKUH for any medical service are protected and recorded by the Department of Health Information Management System (HIMS) with high-end confidentiality.

All patients who present with an act of SH to AKUH undergo a psychiatric evaluation by a psychiatry resident doctor-on-call, and a diagnosis and plan of management are formulated. Medical records of patients with a definite diagnosis of SH, of either sex, aged 11 years and above at the time of admission, were selected from the HIMS, using the departmental internal log and the unique computerized coding. Cases related to “Drug Overdose, Poison Ingestion, Suicide, Attempted Suicide, (Deliberate) Self-Harm, Self-Injury, and Physical Harm” were selected. The study proforma included information related to demographic characteristics, past history of psychiatric or any other medical illnesses, which were collected from medical records. Besides, information related to the method of SH, the intent to die, and prior SH was also extracted. We confirm that the study protocol was approved by the Institutional Ethical Review Committee of the Aga Khan University (approval no: 5133-SON-ERC-17). Since this study included a review of secondary data (file records) and does not involve any human interaction, therefore, Institutional Ethical Review Committee of the Aga Khan University has waived the need for informed consent. Authors confirm that all methods were carried out in accordance with ethical guidelines and regulation of the institution.

All data were analysed using STATA version 14. Quantitative variables were summarized using mean and standard deviation, whereas categorical variables were summarized using frequency and percentages and 95% confidence interval for the total observations, using a complete case analysis. Descriptive analysis for psychiatric and medical history was classified based on intention to die. The Chi-square test of independence was used to determine the association of intention to die with psychiatric and medical illness and prior SH.

## Results

A total of 350 cases of SH that were reported during the years 2013 to 2017 at AKUH were analysed.

### Socio-demographic characteristics

Out of the 350 cases, 68.3% (*n* = 239) were females and 31.7% (*n* = 111) were males. Their ages ranged from 11 to 75 years. More than half of the cases of SH (63%) were in the age group of 20–39 years, followed by adolescents aged 11–19 years (21%). About 46% (*n* = 161) had had no formal education, whereas 5.4% (*n* = 19) were unemployed. Other socio-demographic variables are listed in Table 1.

**Table 1:** Socio-demographic characteristics of self-harm cases (*n* = 350)

Socio-Demographic Characteristics	n (%; 95% CI)
**Age (in years)**	
Adolescents (11–19 years)	72 (20.6; 16.4 – 25.9)
Young adults (20 to 39 years)	221 (63.1; 57.8 – 68.2)
Middle adult (40 to 59 years)	53 (15.4; 11.5 – 19.3)
Older adults (60 years and above)	4 (1.4; 0.3 – 2.0)
**Religion**	
Muslim	306 (87.4; 83.4 – 90.7)
Others	44 (12.6; 9.2 – 16.5)
**Marital Status**	
Single	161 (46.0; 40.6 – 51.3)
Ever Married	189 (54.0; 48.6 – 59.3)
Married	176 (50.3; 44.9 – 55.6)
Divorced/separated	13 (3.7; 1.9 – 6.2)
**Occupation**	
Home maker	131 (37.4; 32.3 -42.7)
Student	101 (28.9; 24.1 – 33.9)
Employed	99 (28.3; 26.3 – 33.3)
Unemployed	19 (5.4; 3.3 – 8.3)
**Educational status**	
No formal education	161 (46.0; 40.5 – 51.3)
Up to secondary	79 (22.6; 18.3 – 27.3)
Above secondary	110 (31.4; 26.6 – 35.5)

### Medical and psychiatric history

Table 2 provides details about the medical and psychiatric history of reported cases classified based on intention to die. In this study, 87% (*n* = 304) of the cases had no history of medical illness. Nearly 72% (*n* = 253) of SH cases had no past psychiatric illness; however, a significant association was found between the past psychiatric illness and SH cases who reported an intent to die. About half (*n* = 45) of the cases with an intent to die had a history of past psychiatric illness, while 47% of cases (with intent to die) were diagnosed with depression at the time of admission (*n* = 46). Almost 80% (*n* = 285) of SH cases had no history of the previous attempt and also reported no intention to die; however, amongst patients with a history of one prior attempt, 35.7% (*n* = 15) had an intention to die, while 39.1% (*n* = 9) had the intention to die with a history of multiple (at least two) prior SH. However, SH cases diagnosed with psychiatric illness were 44.6% (*n* = 156) and were significant with intent status (*p*-value < 0.001). The most common diagnosis was depression.

**Table 2:** Medical and psychiatric history of self-harm cases based on intention to die (*n* = 350)

**Medical / Psychiatric History**	**n (%; 95% CI)**	**No Intent to Die** 253 (72.3)	**Intent to Die** 97 (27.7)	***P*-value**
History of medical illness				0.658
Yes	46 (13.1; 9.7 -17.1)	32 (69.6)	14 (30.4)	
No	304 (86.9; 82.8 – 90.2)	221 (72.7)	83 (27.3)	
Past psychiatric history				< 0.001
Yes	97 (27.7; 23.0 – 32.7)	52 (53.6)	45 (46.4)	
On medications	68 (70.1; 59.9 – 78.9)	34 (50.0)	34 (50.0)	
No medications	29 (29.9; 21.0 -40.0)	18 (62.1)	11 (37.9)	
No	253 (72.3; 67.2—76.9)	201 (79.5)	52 (20.5)	
Prior self-harm				0.066
Yes	65 (18.6; 14.6 – 23.0)	41 (63.1)	24 (36.9)	
Once	42 (64.6; 51.7 – 76.0)	27 (64.3)	15 (35.7)	
Multiple	23 (35.3; 23.9 -48.2)	14 (60.9)	9 (39.1)	
No	285 (81.4; 76.9 – 85.3)	212 (71.4)	73 (25.6)	
Psychiatric diagnosis on assessment				< 0.001
Absent	194 (55.4; 50.5 – 60.7)	163 (84.0)	31 (16.0)	
Present	156 (44.6; 39.2 – 45.9)	90 (57.7)	66 (42.3)	

*P-*value < 0.05 considered significant using Chi-square test of independence

### Method used and reasons for self-harm

Table 3 illustrate details of the methods used and the reason for SH. The most common method used was drug overdose 61.1% (*n* = 214), with benzodiazepines the most common drug used in 57% (*n* = 121) cases. The second most common method used was the ingestion of pesticides 36.6% (*n* = 128). In a few cases, 2.3% (*n* = 8) reported physical harm. A substantial proportion (82%, *n* = 288) reported that the chosen method (drugs and pesticides) was available at home, while 18% (*n* = 62) had bought it, especially with the intention of SH. More than half of the reported reasons for SH were inter-personal relationship conflicts, including marital, in-laws and other familial and close-one relationships. Supplementary Table 1 provides additional details of factors stratified by sex.

**Table 3:** Methods used and reasons for self-harm (*n* = 350)

	**n (%; 95% CI)**
**Method used for current attempt to inflict self-harm**	
Drug overdose	214 (61.1; 55.8 – 65.2)
Ingestion of pesticides	128 (36.6; 89.6 – 97.8)
Harming physically	8 (2.3; 2.5 – 11.6)
**Access to the chosen method**	
From home	288 (82.3; 77.8 – 86.1)
Items available at home	262 (90.7; 87.5 – 94.02)
Prescribed drugs at home	26 (9.3; 5.9 – 12.9)
Brought from outside	62 (17.7; 13.2 – 22.1)
**Reason for attempt to self-harm**	
Interpersonal relationship conflicts	190 (54.3; 48.9 – 59.6)
Financial	14 (4.0; 2.2 – 6.6)
Academic difficulties	17 (4.9; 2.9 – 7.7)
Psychiatric. Illness	20 (5.7; 3.5 – 8.7)
Medical Illness	4 (1.1; 0.3 – 2.9)
Bereavement	6 (1.7; 0.6 – 3.7)
Refuse to reveal	45 (12.9; 9.5 – 16.8)
Mistake	8 (2.3; 0.1 – 1.5)
Multiple issues	46 (13.1; 9.8 – 17.1)

## Discussion

This study provides the characteristics and pattern of individuals who reported with SH in a private tertiary care teaching hospital in Karachi, Pakistan. The current study reported a higher proportion of SH among young adults, and those having a past SH attempt and psychiatric history. A strong association of psychiatric illness and intent to die was evident in this study. The intent to die was reported in more than a quarter of reported cases, and self-poisoning was found as a common method used. Our findings align with earlier studies conducted in Pakistan [7, 13] and from other countries in the region, including Sri Lanka, Malaysia, and India, reporting a peak of SH among people of 20–29 years [14–16]. The probable reasons for the vulnerability of SH behaviour in this age group are recurrent life stressors, pre-morbid psychiatric illnesses, handling difficult personal and interpersonal relationships [17, 18], impulsivity, and maladaptive coping.

Past history of SH is a strong predictor for subsequent SH and/or completed suicide in HIC [19–21]. In contrast, the current study reported less than one-fifth of the cases with a history of one or more SH behaviours. Similar findings from earlier national studies also reported less prevalent psychiatric illness in SH cases [22, 23]. Interpersonal challenges play a crucial role in SH in Pakistan [13, 22, 23]. The studies from HIC find a strong association of past psychiatric illness with SH [21], contrary to this study’s finding. In scoping review from Pakistan, only a small percentage of research has identified mental illness as a risk factor for SH [13]. The possible reason could be underreporting of mental illness due to stigma and inadequate assessment [13]. Another possible reason for the low rate of repeat SH in Asia is that people who self-harm have fewer mental disorders than people who self-harm in the HIC.

Moreover, in the current study, the intention to die was strongly associated with psychiatric illness, consistent with the earlier study [24]. Therefore, psychiatric evaluation and follow-up care are also vital for preventing repeated SH [25]. The current study found that more than a quarter of SH cases had ‘suicide intent’, defined as “the seriousness or intensity of the wish of a patient to terminate his life” (p. 45) [26]. Literature suggests that intent of suicide is a significant predictor of later suicide attempts or completed suicide [27]. However, the presence of mental illness does not predict the actual intent to die in all cases of SH. Our study reported ‘intent to die’ in only a quarter of the cases. This finding is optimistic, indicating that the attempts could have been prevented in a majority of the cases if accessible and affordable psychological support had been available to manage the impulsive SH thoughts and life circumstances [24].

Drug overdose, particularly benzodiazepine and ingestion of pesticides, accounts for more than two-thirds of the total SH cases in this study, is comparable with earlier studies from Pakistan [7, 28]. Regarding the use of pesticides, our study is in consort with other studies conducted in China and India [29–31]. In India, hanging is reported as the most common method of suicide, followed by pesticide ingestion [32]. In comparison, none of the SH cases was reported by hanging in this study. The probable reason could be that this study focused on SH patterns rather than suicide as an outcome. For instance, in Pakistan, hanging was also reported as the most common method of suicide [31].

Moreover, benzodiazepines are reported as the most common medication used in SH in national and regional studies [33–37]. A notable finding was that the access to the mean was available at home in most of the reported cases. A plausible reason could be the over-the-counter (OTC) and low-cost availability of benzodiazepines in countries such as Pakistan [28].

The findings of this study have limited generalizability since the data are reported from a single private institution in Karachi, Pakistan and are limited to the cases that require medical attention and may afford the treatment cost. Thus, cautious generalization of findings to all socio-economic strata is suggested. Additionally, ‘intent to die’ was based on clinical assessment rather than a standard or valid tool. We also need to consider the sensitivity of SH in the context of a conservative Islamic society like Pakistan, which strongly condemns such acts. Thus, the intent to die could be underreported.

## Conclusion and recommendations

This study has provided a noteworthy finding for characteristics and patterns of individuals who have self-harmed in Karachi, Pakistan. Past Psychiatric history and repeated SH were less commonly reported in this study. A higher proportion of young adults and intent to die was reported in nearly one-quarter of the total cases. The most common methods used were drug overdose and insecticides. Interpersonal relationship conflicts were the most prevailing reason for SH.

Government regulations and bans on pesticides can be effective measures learning from the example of Sri Lanka [38] and India [32]. Such measures are also considered cost-effective and affordable strategies for suicide prevention [39, 40]. Prevention of SH needs to be considered both for the general population and individuals at risk. Integrating a holistic psychosocial assessment needs to be considered in primary care for prevention [41]. We recommend setting up surveillance systems to collect data prospectively on SH [34, 42] from both health facility and community settings for a comprehensive understanding of this phenomenon. Longitudinal studies are required to understand better the complex factors associated with SH cases.

## Supplementary Information

**Additional file 1:** **Supplementarytable 1.** Reasons for self-harm (*n* = 350).

## Data Availability

The datasets generated and/or analysed during this study are not publicly available due to confidential information of patients but are available from the corresponding author on reasonable request.
