# Supplementary material for: Characteristics and patterns of individuals who have self-harmed: a retrospective descriptive study from Karachi, Pakistan
Source: BMC Psychiatry. 2022 May 31;22:367. doi: 10.1186/s12888-022-04018-7 (PMC9158237; doi:10.1186/s12888-022-04018-7)
Supplement: Supplementary file 1 — Additional file 1: Supplementarytable 1. Reasons for self-harm (n = 350). [file 12888_2022_4018_MOESM1_ESM.docx]

Supplementary table 1: Reasons for self-harm (n = 350)

|  | **n (%)** | **Male**  111 (31.7) | **Female**  239 (68.3) |
| --- | --- | --- | --- |
| **Reason for attempt to self-harm**  Interpersonal relationship conflicts  Financial  Academic difficulties  Psychiatric. Illness  Medical Illness  Bereavement  Refuse to reveal  Mistake  Multiple issues | 190 (54.3)  14 (4.0)  17 (4.9)  20 (5.7)  4 (1.1)  6 (1.7)  45 (12.9)  8 (2.3)  46 (13.1) | 60 (31.6)  9 (8.1)  4 (3.6)  5 (4.5)  -  3 (2.7)  11 (9.9)  3 (2.7)  16 (14.4) | 130 (68.4)  5 (2.09)  13 (5.44)  15 (6.28)  4 (1.67)  3 (1.26)  34 (14.23)  5 (2.09)  30 (12.6) |
